# Physiotherapy Management Focusing on Early Mobilisation in Complex Pelvic and Pott’s Fracture

**DOI:** 10.7759/cureus.49525

**Published:** 2023-11-27

**Authors:** Deepali S Patil, Vaishnavi M Thakre, Nikita Gangwani

**Affiliations:** 1 Musculoskeletal Physiotherapy, Ravi Nair Physiotherapy College, Datta Meghe Institute of Higher Education and Research, Wardha, IND

**Keywords:** pott's fracture, bimalleolar ankle fracture, orif, early mobilization, physiotherapy, pelvic

## Abstract

Pelvic fractures (PFs), involving the disruption of the bony structures in the pelvic region, are complex injuries often associated with high-energy trauma. Such fractures can significantly impact a patient's mobility and overall quality of life. Concurrently, fractures of the malleoli, specifically the lateral and medial aspects of the ankle, are common lower extremity injuries that can result from various mechanisms, including twisting or direct trauma. This case report presents the multidisciplinary approach employed in the successful treatment of a 26-year-old male patient with a rare combination of PFs involving both anterior and posterior columns, along with lateral and medial malleolus fractures. The patient underwent a series of surgical interventions to stabilise the fractures, followed by a tailored physiotherapy management plan. The report discusses the postoperative care strategies and the crucial role of physiotherapy in the rehabilitation process.

## Introduction

Approximately 10% of all blunt trauma admissions are related to injuries in the pelvic region [1]. The pelvic structure is separated into the anterior and posterior parts, which are known as the pelvic girdle and pelvic spine, respectively [2,3]. It's important to note that both-column fractures of the acetabulum typically result from high-energy trauma (87.4%), and notably, 61.5% of the affected patients sustain these injuries in road traffic accidents [4]. Some of the most common surgical procedures include minimally invasive plate osteosynthesis (MIPO), external fixation, open reduction and internal fixation (ORIF), and intramedullary nailing [5]. Physiotherapists have a key part in the rehabilitation of individuals who have suffered pelvic fractures (PFs). They strive to alleviate pain, strengthen muscles, improve joint mobility, promote function, and address pelvic dysfunction [6].

The ankle joint is a type of synovial hinge joint that is formed through the intricate articulation of three distinct bones, which includes the talus and the distal tibia and fibula and operates within a single plane, facilitating movements such as plantar flexion and dorsiflexion [7]. Within the ankle joint, there are three essential components known as malleoli, which are crucial for stability and movement control. It includes the lateral malleolus located at the fibular distal end, the medial malleolus situated at the inner aspect of the distal tibia, and the posterior malleolus. The malleolar fracture can be unimalleolar, bimalleolar or trimalleolar. The bimalleolar fracture is also known as Pott’s fracture [8]. Ankle joint injuries are common. Males are more prevalent than females to sustain physeal ankle fractures, which account for around 60% of cases during athletic activity [9]. Management for these fractures focuses on restoring alignment and joint stability in order to lessen the probability of post-traumatic ankle arthritis [10,11]. In accordance with the specific fracture type, they can be managed conservatively or surgically, usually accompanied by an immobilisation period. Early rehabilitation is strongly recommended in the management of ankle joint injuries [12].

It is widely assumed that physical therapy restores the decline in fatigue resistance, functioning, and performance of muscle caused by immobilisation and the trauma itself [13]. As a result, we devised a physiotherapy programme centred on early rehabilitation and sensorimotor retraining to assist these patients in improving their motor control, muscle strength, and gait.

## Case presentation

Patient information

We present a case of a 26-year-old male who met an accident while riding a bicycle and got thrashed by a petrol truck. Then, he was immediately taken to a nearby hospital, where basic medical management was provided. Due to a lack of facilities, he was further referred to Acharya Vinoba Bhave Rural Hospital (AVBRH), where he was admitted to the casualty ward and later shifted to the neuro-intensive care unit (ICU). After consulting an orthopaedic surgeon, the patient underwent investigations like a CT scan and X-ray, which revealed a complex pelvic and bimalleolar fracture on the left side. Then, the patient underwent ORIF with tension band wiring for medial malleolus fracture of the left side, ORIF with plate osteosynthesis for fracture lateral malleolus and surgical correction of pelvic bone fracture for posterior column fixation of the left side. Post-operatively, the patient experienced pain and reduced mobility in the left lower limb, for which physiotherapy was commenced in October 2023.

Clinical findings

Prior to commencing the examination, the patient's informed consent was obtained, following which a thorough examination was conducted. The patient was hemodynamically stable. At the examination, he assumed a supine-lying position with a 30^o^ elevation of the head end and the knees and ankles supported using pillows. Physically, the patient presented a mesomorphic physique. The pain intensity was rated as 4/10 on rest and 7/10 on activity according to the numerical pain rating scale (NPRS), which was dull aching on the operated site. Mild swelling was present around the ankle joint, and tenderness was grade 2 according the tenderness grading scale that is patient winces due to pain. The movements at the left ankle and hip were painful. The hip and ankle musculature strength and range of motion (ROM) were reduced. The straight-leg raise test was unable to be executed due to the pain and fracture.

Diagnostic assessment

**Figure 1:**
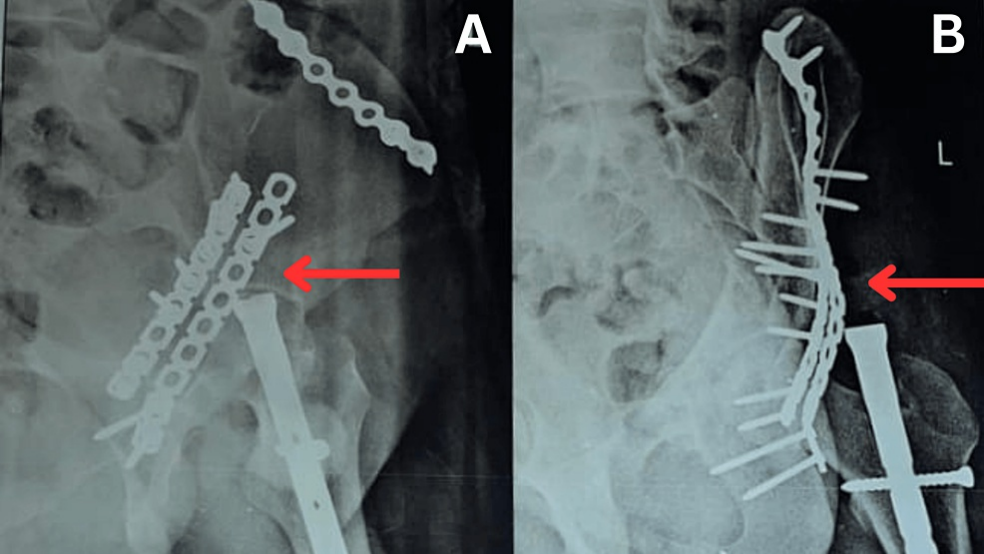
X-ray showing post-operative anterior column PF of left side Figure 1A: Arrow shows anterior ring stabilisation done with plates and screws (anterior-posterior view) Figure 1B: Arrow shows anterior ring stabilisation done with plates and screws (lateral view) PF: Pelvic fracture

**Figure 2:**
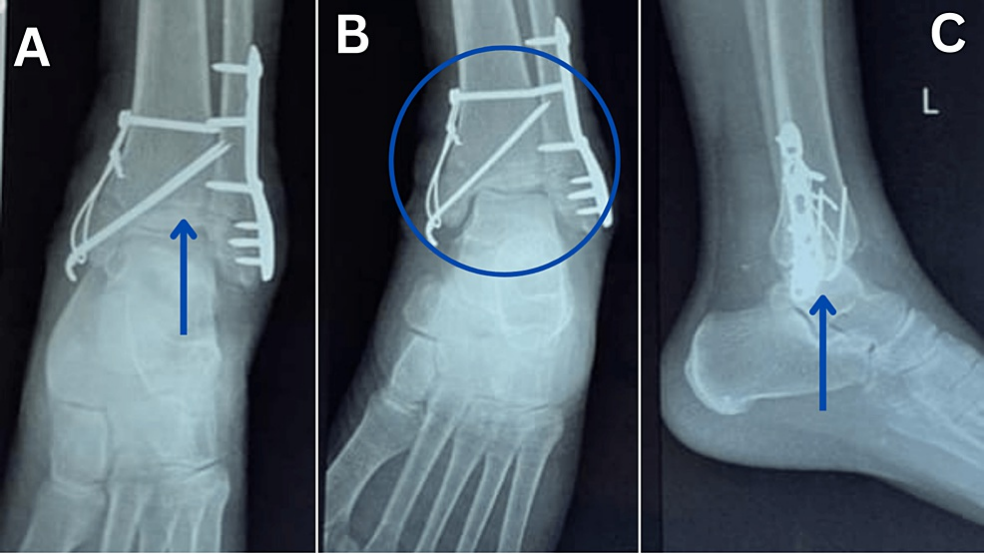
X-ray of post-operative medial and lateral malleolus fracture (left side) Figure 2A and 2B: Arrow and circle show ORIF done with nailing and plating in (anterior-posterior view) Figure 2C: Arrow show ORIF done with nailing and plating (lateral view) ORIF: Open reduction and internal fixation

The X-ray reports revealed ORIF with tension band wiring for the medial malleolus fracture of the left side, ORIF with plate osteosynthesis for fracture lateral malleolus (Figure 1A-1B) and surgical correction of pelvic bone fracture for fixation of the posterior column on the left side (Figure 2A-2C).

Physiotherapy intervention

**Figure 3:**
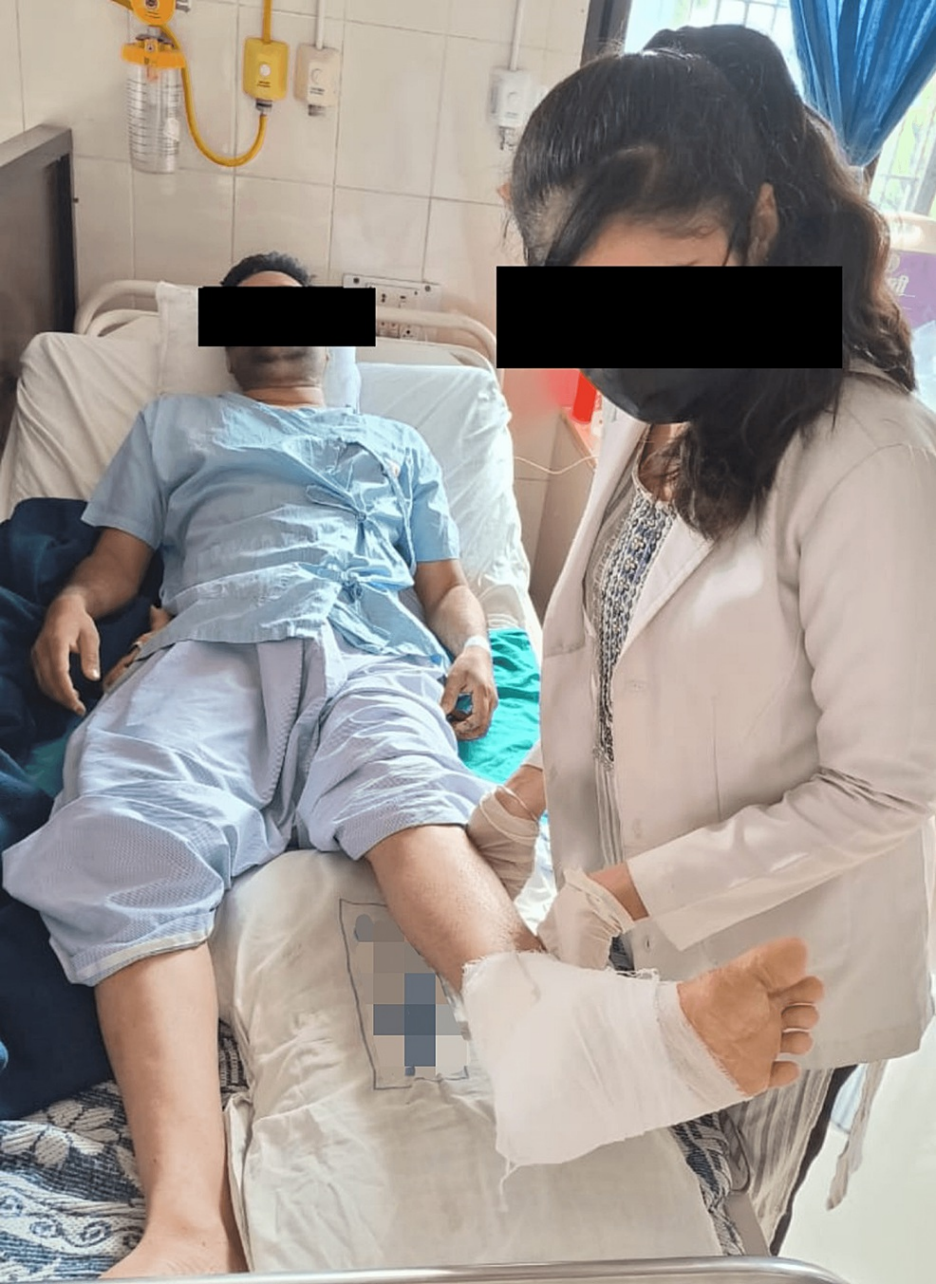
Patient performing active assisted SLR SLR: Straight leg raise

**Figure 4:**
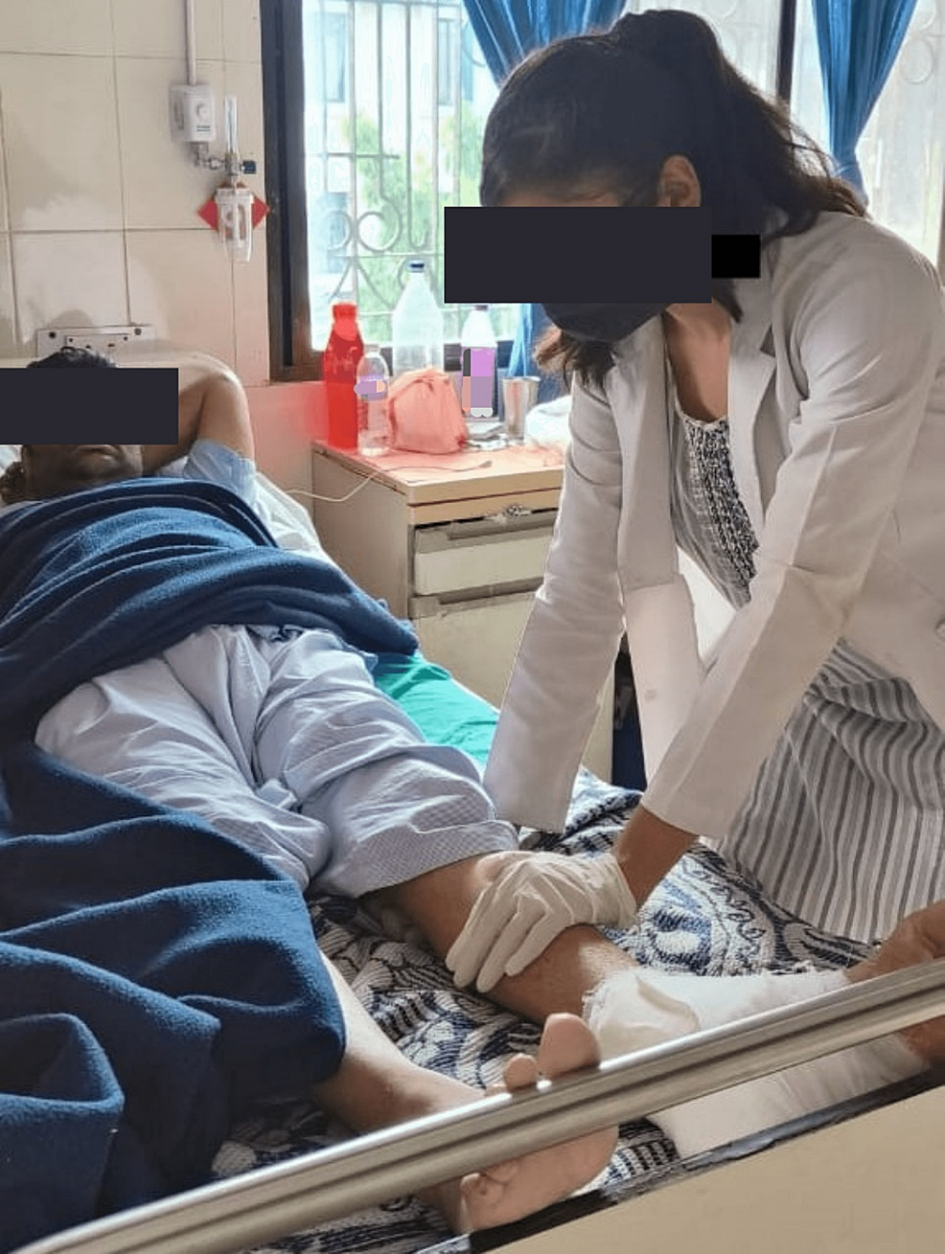
Patient undergoing isometric quadriceps exercises

An organised physical therapy protocol was started for four weeks. The patient was well-explained regarding protocol and was supervised throughout the rehabilitation (Table 1, Figures 3,4).

**Table 1:** Physiotherapy intervention NA: Not applicable, Reps: Repetitions, ROM: Range of motion, VMO: Vastus medialis oblique

Goal	Intervention	Frequency	Progression
Patient and family education	Patient and along with his family was well-explained regarding his condition and was told about the importance of physiotherapy intervention.	NA	Home programme explained
Prevention of any vascular and pulmonary complication	Ankle pumps	20 reps x 1 set, Thrice daily	NA
	Deep breathing exercises	10 reps x 1 set, Twice daily	
To reduce pain	Cryotherapy	7 minutes, Twice daily	NA
Restore mobility of hip and ankle joints	Assisted ROM exercises of the involved hip and knee (Heel slides, straight leg raises)	10 reps x 1 set, Thrice daily	Active ROM exercises of the involved hip & knee
Prevent the knee and hip muscle postoperative reflex inhibition	Low-intensity isometric exercises of the hip and knee musculature of the operated extremity	20 reps with 10 seconds hold x 1 set	VMO strengthening with bolster
To improve trunk and pelvic control	Core stability exercises (pelvic tilts, pelvic bridging)	10 reps x 1 set, Twice daily	Full push-ups in prone lying
To increase muscular strength of lower limb	Isometric exercise of quadriceps, hamstring and gastro-soleus muscle	10 reps with 5 seconds hold x 1 set	Non-weight-bearing and weight- bearing dynamic resistance exercises
To gain static and dynamic balance	Balance retraining in parallel bars	Twice daily	Gait training with crutches progressing to independent ambulation

Follow-up and outcomes

A follow-up was carried out once per week after four weeks of physical therapy. Tables 2,3,4 show pre- and post-treatment outcomes for various parameters. 

**Table 2:** Pre- and post-intervention ROM ROM: Range of motion

Muscles	Pre- intervention	Post- intervention
Hip flexion	20^0^	70^0^
Knee flexion	15^0^	100^0^
Ankle dorsiflexion	12^0^	20^0^

**Table 3:** Pre- and post-intervention MMT 0: No contractions; 1: Flickering of contractions; 2: Full ROM with gravity eliminated; 3: Full ROM against gravity; 4: Full ROM against moderate resistance; 5: Full ROM against maximum resistance MMT: Manual muscle testing; ROM: Range of motion

Muscles	Right	Left
Hip flexors	2	5
Hip extensors	2	4
Hip abductors	2	4
Knee flexors	2	5
Ankle planterflexors	3	4
Ankle dorsiflexors	2	5

**Table 4:** Outcome measures NPRS: Numerical pain rating scale

Scale	1^st^ week	4^th^ week
NPRS	7	1
Functional Independence Measure	20/126	100/126
Lower Extremity Functional Scale	15/80	65/80

## Discussion

In this particular case, the patient underwent surgery to address both a PF and a Pott’s fracture of the ankle. Physiotherapy was initiated as a preventive measure against further complications and to facilitate early ambulation. The physiotherapy interventions were carefully planned with the primary goals of complication prevention and promoting early mobility. Following a PF, a complete rehabilitation regimen is necessary, and a reliable and appropriate clinical assessment provides optimal treatment of complications. Even though there is no high-quality data for rehabilitative strategies after PF in regards to the modality of therapy and duration and long-term functional outcomes, early multidisciplinary intervention is strongly suggested to promote recovery from PF [14,15].

According to Fokmare et al., the three weeks of physical therapy regimen encompassing proprioceptive training, mulligan's mobilisation with ultrasound therapy, gait training, mobilisation, and strength training, which enhanced the individual's overall functioning, strength and the range of their ankle dorsiflexion in the patients having chronic post-operative trimalleolar ankle fracture [16,17]. The malleolar fracture, which provides good visualisation, proper fracture restoration, and anatomical reduction, can result in reducing complications and improving the final prognosis [18]. When compared to patients without weight-bearing for six weeks, early weight-bearing (EWB) patients were able to tolerate full weight-bearing more rapidly. Additionally, there were no adverse impacts on hospital stays, pain levels, or the amount of time it took to return to work when compared with EWB [19,20].

The central aim of this case report was to underscore the critical importance of implementing a well-structured early treatment protocol and comprehensive rehabilitation programme for a young adult who had suffered multiple fractures in their lower extremities. This report sought to bring attention to the significance of early intervention and a holistic approach to care in cases of lower extremity fractures in this specific demographic.

## Conclusions

In conclusion, this case report highlights the successful management of a complex musculoskeletal trauma involving pelvic anterior and posterior column fractures along with a bimalleolar ankle fracture. Early initiation of physiotherapy played a crucial role in the patient's rehabilitation, ensuring optimal functional recovery and minimising long-term complications. This case emphasizes the importance of a multidisciplinary approach, combining surgical expertise and comprehensive physiotherapeutic interventions, in achieving favourable outcomes for patients with such intricate injuries. It also underscores the significance of timely intervention, meticulous surgical techniques, and personalised rehabilitation programmes in restoring patients' mobility and overall quality of life after severe musculoskeletal trauma. The limitation of this case study is its need for more generalisability, as the care given to one patient might not result in the same outcome in another.
